# Tyrosine kinase inhibitors, ionic currents, and cardiac arrhythmia

**DOI:** 10.3389/fonc.2023.1218821

**Published:** 2023-07-24

**Authors:** Chi-Hao Hu, Sheng-Nan Wu, Edmund Cheung So

**Affiliations:** ^1^ Department of Physiology, National Cheng Kung University Medical College, Tainan, Taiwan; ^2^ School of Medicine, National Sun Yat-sen University College of Medicine, Kaohsiung, Taiwan; ^3^ Department of Education and Research, An Nan Hospital, China Medical University, Tainan, Taiwan; ^4^ Department of Anesthesia, An Nan Hospital, China Medical University, Tainan, Taiwan

**Keywords:** tyrosine kinase inhibitor, ion channels, erg-mediated K+ channel, QTc interval, cardiac arrhythmia

Recently, we have read a very interesting paper entitled “The association between tyrosine kinase inhibitors and fatal arrhythmia in patients with non-small lung cancer in Taiwan” by Chang et al., published in your journal, Frontiers in Oncology. In their study conducted in Taiwan, it was observed that there is a potential correlation between the use of tyrosine kinase inhibitors and fatal cardiac arrhythmias in patients with non-small cell lung cancer. Therefore, we would like to express some related opinions about the paper to provide reference to the readers.

Indeed, many compounds known to suppress the activity of tyrosine kinases in various types of neoplastic cells have been increasing used in clinical practices to treat various types of advance cancers. However, these drugs are recognized to exert inhibitory effects on ion channels in the cell membrane, such as erg-mediated K^+^ channels, which can potentially lead to cardiac arrhythmias. Therefore, regarding the use of tyrosine kinase inhibitors, we may discuss this from two perspectives: clinical and basic research.

## First, on the level of clinical research

1. There have been many different tyrosine kinase inhibitors (such as lapatinib and sorafenib) approved for the treatment of various serious cancers, including non-small cell lung carcinoma (1, 2). With appropriate administration, there is evidence that many good treatment outcomes can occur, bringing many positive implications for disease treatment and prognosis. However, the use of these drugs can sometimes cause a lengthening in cardiac action potential or prolongation of the QTc interval on the electrocardiogram, leading to severe Torsade Pointe arrhythmia (1). As a result, there may be an increased risk of sudden death due to cardiac causes. This is an area where healthcare professionals and even family members need to be more aware and understanding. On the other hand, encouraging patients to carry portable electrocardiogram monitors with them may be an important tool for early detection.
2. In addition to tyrosine kinase inhibitors, many cyclin-dependent kinase (CDK) inhibitors (such as ribociclib) have also shown good treatment outcomes for patients with various severe cancers. However, there are also frequent occurrences of QTc interval prolongation issues (3). It is therefore important to note that if these different anti-cancer drugs are used in combination, there is a high likelihood of significantly increasing the occurrence of cardiac arrhythmias.
3. Of note, in patients with concomitant atrial fibrillation, the administration of tyrosine kinase inhibitors can block potassium ion currents, similar to the effects of quinidine or procainamide, causing a shortening of the atrioventricular node refractoriness and leading to rapid ventricular response. This aspect should also be given special attention in clinical practice to prevent unexpected events.
4. Many cancer patients receiving tyrosine kinase inhibitors may experience changes in their blood electrolyte levels. For example, changes in potassium ion concentration can affect the QTc interval on electrocardiogram, leading to arrhythmias (4, 5). Therefore, the concentration of potassium ions in the blood can also affect the effect of tyrosine kinase inhibitors on QTc intervals.

## Second, on the level of basic academic research

1. Although the blockade of hERG K^+^ currents by tyrosine kinase inhibitors may be one of the reasons for QTc interval prolongation induced by these drugs (6, 7), inhibition of other potassium currents can also lead to lengthening of the cardiac action potential and concurrent prolongation of the QTc interval (7). Therefore, in drug development, in addition to studying the degree of inhibition of tyrosine kinase activity by different drugs, it is necessary to conduct more in-depth research on the mechanisms of action of these new anti-neoplastic drugs on various ion currents on the cell membrane, such as current intensity or kinetics of opening or closing, to reduce the risk of QTc interval prolongation after their administration (8).
2. In addition to hERG K^+^ currents, blocking slowly activating delayed-rectifier K^+^ currents (also known as KCNQ1 or K_V_7.1 current) can also lead to QTc interval prolongation. Moreover, the current inactivation of certain delayed rectifier K^+^ currents (especially K_V_2.1 or K_V_3.1 current) can cause QTc interval prolongation, and even polymorphic ventricular tachycardia (9). In addition, some drugs that activate voltage-gated sodium currents can also cause QTc interval prolongation (10, 11).
3. Tyrosine kinase inhibitors have IC_50_ values in the nM range for tyrosine kinase inhibition (11), while the doses that cause inhibition of K^+^ currents by tyrosine kinase inhibitors are in the µM range (7, 8). The difference between the two is about 100-1000 times. Moreover, for drugs like zanubrutinib, the pharmacokinetics show a maximum blood concentration of about 400 ng/ml (approximately 0.85 µM) (12). Therefore, the impact on membrane K^+^ currents seems to be minimal.
4. Finally, whether the inhibition of these membrane ion currents directly or indirectly participate in the suppression of tyrosine kinase activity is also an important subject for further research.

**Figure 1:**
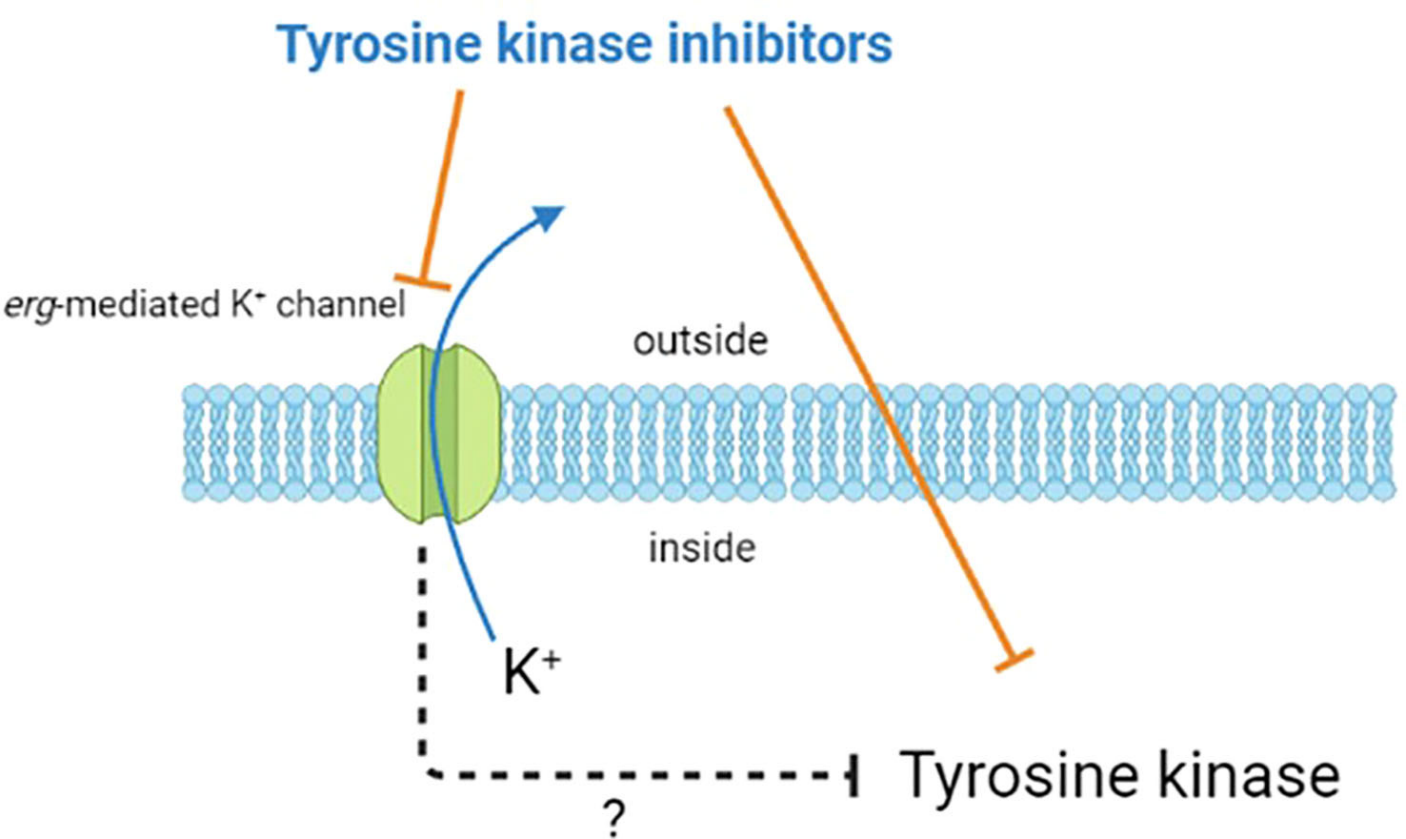
Diagram illustrating the potential effects of tyrosine kinase inhibitors. Besides inhibiting the activity of tyrosine kinases in cells, they may also block erg-mediated K^+^ channels. However, the indirect impact of this channel blockade on the activity of tyrosine kinases is current unclear and require further research.

In summary, as shown in **Figure 1**, apart from its inhibition of tyrosine kinases, the compounds known to suppress the activity of tyrosine kinases may affect the activity of *erg*-mediated K^+^ channels. However, how the inhibition of these ionic channels can indirectly alter the activity of tyrosine kinases remains to be further investigated. It is hoped that the understanding and research in this area can be further enhanced, while providing better assistance for the care and treatment of patients.

## Author contributions

S-NW and ES drafted the manuscript. C-HH prepared the format for submission and help to review and polish the English wordings. All authors contributed to the article and approved the submitted version.
